# Supplementary material for: An efficient approach for broadband sound absorption using periodic multi-layer solid inclusions in acoustic coatings
Source: PLoS One. 2024 Nov 21;19(11):e0311745. doi: 10.1371/journal.pone.0311745 (PMC11581354; doi:10.1371/journal.pone.0311745)
Supplement: Attachment — Submitted filename: Response to Reviewers.doc [file pone.0311745.s003.doc]

Dear Editor

Thank you very much for your letter dated Jan 24, 2024, in which you sent us the reviewers' comments on our paper entitled “An efficient analytical model for acoustic coatings with multi-layer solid inclusions for broadband sound absorption (PONE-D-23-43201)”. We would also like to thank the reviewers for their valuable comments and suggestions.

According to the comments and suggestions, we have revised the manuscript. A summary of the changes is as follows:

Reviewers' comments:

Reviewer's Responses to Questions

Comments to the Author

1. Is the manuscript technically sound, and do the data support the conclusions?

The manuscript must describe a technically sound piece of scientific research with data that supports the conclusions. Experiments must have been conducted rigorously, with appropriate controls, replication, and sample sizes. The conclusions must be drawn appropriately based on the data presented.

Reviewer #1: Partly

Reviewer #2: Yes

The two authoritative literatures recently published on underwater sound absorption of periodic structures have not been experimentally studied [1-2], and according to a 2018 paper, strongly coupled systems are more sensitive to changes in boundary conditions. Therefore, the author speculates that there may be a significant difference between experimental and theoretical results. Therefore, this literature did not conduct experiments. For non-periodic structures, sound absorption can be measured in the underwater impedance tube equipment [3].

[1] Fang, X., Yin, X., Wu, J., Li, Y., Li, H., Wang, W., Li, Y., & Wu, W. (2023). Underwater metagratings for sub-kilohertz low frequency and broadband sound absorption. International Journal of Mechanical Sciences.

[2] Hu, N., Jin, J., Peng, W., Zhang, Z., & Hu, H. (2024). Liquid-solid synergistic mechanism sound absorption for underwater anechoic coating. International Journal of Mechanical Sciences.

[3] Zhang, H., Shi, D., Zha, S., & Wang, Q. (2018). A simple first-order shear deformation theory for vibro-acoustic analysis of the laminated rectangular fluid-structure coupling system. Composite Structures.

2. Has the statistical analysis been performed appropriately and rigorously?

Reviewer #1: N/A

Reviewer #2: No

This article does not involve statistical analysis issues, and the relevant results are converged. It should be noted that two conditions need to be strictly met to adopt the analytical model ($\omega_{0}r/c_{l}$<<1 and $\omega_{0}a/c_{l}$<<1 where cl denotes the longitudinal speed in the PDMS) [1,2].

[1] Fang, X., Yin, X., Wu, J., Li, Y., Li, H., Wang, W., Li, Y., & Wu, W. (2023). Underwater metagratings for sub-kilohertz low frequency and broadband sound absorption. International Journal of Mechanical Sciences.

[2] G.S. Sharma, A. Skvortsov, I. MacGillivray, N. Kessissoglou (2019). Acoustic performance of periodic steel cylinders embedded in a viscoelastic medium. Journal of Sound and Vibration, 443 652-665.

3. Have the authors made all data underlying the findings in their manuscript fully available?

The PLOS Data policy requires authors to make all data underlying the findings described in their manuscript fully available without restriction, with rare exception (please refer to the Data Availability Statement in the manuscript PDF file). The data should be provided as part of the manuscript or its supporting information, or deposited to a public repository. For example, in addition to summary statistics, the data points behind means, medians and variance measures should be available. If there are restrictions on publicly sharing data—e.g. participant privacy or use of data from a third party—those must be specified.

Reviewer #1: No

Reviewer #2: No

The authors have made all data underlying the findings in their manuscript fully available in supplementary materials.

4. Is the manuscript presented in an intelligible fashion and written in standard English?

PLOS ONE does not copyedit accepted manuscripts, so the language in submitted articles must be clear, correct, and unambiguous. Any typographical or grammatical errors should be corrected at revision, so please note any specific errors here.

Reviewer #1: No

Reviewer #2: Yes

After our repeated checks, we have made every effort to improve our article. All modifications have been marked in the article.

5. Review Comments to the Author

Please use the space provided to explain your answers to the questions above. You may also include additional comments for the author, including concerns about dual publication, research ethics, or publication ethics. (Please upload your review as an attachment if it exceeds 20,000 characters)

The questions above are explained.

Reviewer #1: The manuscript involves the theoretical and numerical examination of the acoustic performance of acoustic coatings containing multi-layers of solid inclusions. Overall, the quality of the manuscript needs some significant improvement. Mathematical details are needed. The manuscript can be considered for publication after the following comments are well addressed.

Major comments:

1. Where does Eq. (1) come from? Please explain the calculation of Eq. (1) or add relevant references if there is any.

The relevant reference is added. These formulas 1-13 are all cited from the same literature (Acoustic performance of periodic steel cylinders embedded in a viscoelastic medium) [13].

[13] G.S. Sharma, A. Skvortsov, I. MacGillivray, N. Kessissoglou (2019). Acoustic performance of periodic steel cylinders embedded in a viscoelastic medium. Journal of Sound and Vibration, 443 652-665.

2. Again, how do the authors obtain Eqs. (2-6)? Please provide clear derivations.

The relevant reference is added.

3. Provide the details about obtaining Eqs. (10) and (11).

The relevant reference is added.

4. Second row on Page 10, what does “the same relationship” mean?

The sentence has been changed as: “Equations 12 and 13 can also be used to obtain the impedance and wave number of the homogenous layer.”.

5. The authors should provide the mathematical derivations of the transfer matrix method and detailed settings for the corresponding FEM simulations.

The transfer matrix method and finite element method are represented in Appendixes A and B in supplementary materials.

“**Appendix A. Genetic Algorithm**

The genetic algorithm is used to determine the geometrical parameters of the multilayer structure with broadband sound absorption. The assumed total thickness of the structure is 60 mm. The parameters that are primarily optimized contain *r*_1,_ *r*_2_, *a*_1_= *a*_2_=*a*, *t*_1_, *t*_2_. The definition of the optimization vector and constraint range for these parameters is as following

(s1)

,,,,.

The determination of the objective function involves finding the maximum value of the average absorption coefficient within a designated frequency range (*f*_min_, *f*_max_), is denoted as

(s2)

where ∆*f*= *f*_min_-*f*_max_.

**Appendix B. Transfer matrix method**

After the effective density, wave number, and thickness of each layer are achieved, the surface impedance of each layer of the structure can be derived utilizing the transfer matrix method , which relates the sound pressure and velocity between the water (*P_w_*,*V_w_*) and air domain (*P_a_*,*V_a_*) is

(s3)

in which

(s4)

Here *s* = 1, 2, …, *s* represents the serial number of the transfer matrices (s = 6 in Fig. 1(b)). *k_s_* (, where *c_l_* stands for longitudinal wave speed.) and *h_s_* stand for the wave number and the transmission length of the *s* th layer medium. Therefore the reflection and transmission coefficients can be calculate by

(s5)

(s6)

where *Z_in_* and *Z_tr_* are the impedance of the incident (water) and transmission (air) domains, respectively. Then the absorption coefficient can be computed as

(s7)

**Appendix C.** **Finite Element Simulation**

To verify the correctness of the proposed algorithm, a finite element simulation in the frequency domain by using the COMSOL Multiphysics is carried out. Only one unit cell is considered due to the periodicity. As shown in fig. S1, the water and air domain are imitated by the acoustic module, and the PDMS domain and steel are simulated by the solid mechanical module. Periodic boundary conditions are applied on the upper and lower boundaries, while the perfect matching layer (PML) is used to eliminate reflection on the left and right boundaries. The structural-acoustic interaction is adopted between the fluid domain and solid domain. The background pressure field (water domain) simulates the incident acoustic wave of unity pressure amplitude. The entire region is divided into triangular meshes, the size of which is controlled by the physical field. The reflected and transmitted pressures can be obtained at the interface between the fluid and solid media on the incidence and transmission sides, respectively .

Fig. S1 Simulation settings for the single-layer structure in COMSOL Multiphysics.

**References**

1.	Cao Z, Li X, Liu B. Broadband sound absorption of a hybrid absorber with shunt loudspeaker and perforated plates. Applied Acoustics. 2023;203:109185.

2.	Li X, Liu B, Chang D. An acoustic impedance structure consisting of perforated panel resonator and porous material for low-to-mid frequency sound absorption. Applied Acoustics. 2021;180:108069.

3.	Li X, Cao Z, Li Z, Liu B. Sound absorption of a shunt loudspeaker on a perforated plate. Applied Acoustics. 2022;193:108776.

4.	Sharma GS, Skvortsov A, MacGillivray I, Kessissoglou N. Acoustic performance of gratings of cylindrical voids in a soft elastic medium with a steel backing. The Journal of the Acoustical Society of America. 2017;141:4694-704.

5.	Fang X, Yin X, Wu J, Li Y, Li H, Wang W, Li Y, Wu W. Underwater metagratings for sub-kilohertz low frequency and broadband sound absorption. International Journal of Mechanical Sciences. 2023;260:108630.

6.	Sharma GS, Skvortsov A, MacGillivray I, Kessissoglou N. Sound absorption by rubber coatings with periodic voids and hard inclusions. Applied Acoustics. 2019;143:200-10.”

6. What are the detailed properties of the genetic algorithm (GA), such as procedures, parameters etc, used in this manuscript?

The genetic algorithm (GA) is represented in Appendix C in supplementary materials.

7. Even with the GA, the results are not impressive at all, especially for low frequencies, compared with the reference "A Fano-based acoustic metamaterial for ultra-broadband sound barriers", Proc. R. Soc. A (2021) 477: 20210024. Please clarify the significant contribution of this work.

Distinct from previous studies that limit the configuration of anechoic coatings to a given law and inefficiently alter numerous geometric parameters for optimal acoustic performance, the metagrating comprising multi-layer hard inclusions with random periods is adopted to precisely modulate the surface impedance in the target spectrum, leading to effective absorption performance in broadband. Our results will contribute to designing highly load-bearing underwater absorbers to improve the stealth performance of vehicles.

8. Acoustic metamaterials have also been extensively investigated to realize sound transmission loss and absorption. To make the introduction part complete, the authors are recommended to add some discussions about the references (1) Proc. R. Soc. A (2021) 477: 20210024 and (2) Applied Physics Letters (2021) 118, 184101.

These two references have already been cited in this paper.

Minor comment: the language needs some significant improvement.

After our repeated checks, we have made every effort to improve our article. All modifications have been marked in the article.

Reviewer #2: In this manuscript, the authors provided an analytical model for acoustic coatings with multi-layer solid inclusions for broadband sound absorption. In order to obtain better sound absorption performance, the genetic algorithm is used to optimize the two-layer hard inclusions of acoustic coatings. The work has some reference values. However, the innovation points could not find in current version. What is the main academic challenges those solved by this work? Intuitively speaking, this manuscript lacks broad interest, intuitive innovation, and necessary experimental measurement results, making it not suitable for comprehensive journals but more suitable for specialized acoustic journals.

The two authoritative literatures recently published on underwater sound absorption of periodic structures have not been experimentally studied [1-2], and according to a 2018 paper, strongly coupled systems are more sensitive to changes in boundary conditions. Therefore, the author speculates that there may be a significant difference between experimental and theoretical results. On the other hand, the author has no sufficient funds for experimental research. So this literature did not conduct any experiments. For non periodic structures, sound absorption can be measured in the underwater impedance tube equipment [3].

[1] Fang, X., Yin, X., Wu, J., Li, Y., Li, H., Wang, W., Li, Y., & Wu, W. (2023). Underwater metagratings for sub-kilohertz low frequency and broadband sound absorption. International Journal of Mechanical Sciences.

[2] Hu, N., Jin, J., Peng, W., Zhang, Z., & Hu, H. (2024). Liquid-solid synergistic mechanism sound absorption for underwater anechoic coating. International Journal of Mechanical Sciences.

[3] Zhang, H., Shi, D., Zha, S., & Wang, Q. (2018). A simple first-order shear deformation theory for vibro-acoustic analysis of the laminated rectangular fluid-structure coupling system. Composite Structures.
