# Supplementary material for: An efficient approach for broadband sound absorption using periodic multi-layer solid inclusions in acoustic coatings
Source: PLoS One. 2024 Nov 21;19(11):e0311745. doi: 10.1371/journal.pone.0311745 (PMC11581354; doi:10.1371/journal.pone.0311745)
Supplement: Attachment — Submitted filename: Response to Reviewers.doc [file pone.0311745.s004.doc]

Dear Editor

Thank you very much for your letter dated March 26, 2024, in which you sent us the reviewers' comments on our paper entitled “An efficient analytical model for acoustic coatings with multi-layer solid inclusions for broadband sound absorption (PONE-D-23-43201R1)”. We would also like to thank the reviewers for their valuable comments and suggestions.

According to the comments and suggestions, we have revised the manuscript. A summary of the changes is as follows:

Reviewers' comments:

Reviewer's Responses to Questions

Comments to the Author

1. If the authors have adequately addressed your comments raised in a previous round of review and you feel that this manuscript is now acceptable for publication, you may indicate that here to bypass the “Comments to the Author” section, enter your conflict of interest statement in the “Confidential to Editor” section, and submit your "Accept" recommendation.

Reviewer #3: All comments have been addressed

Reviewer #4: (No Response)

After our repeated checks, we have made every effort to improve our article. All modifications have been marked in the article.

2. Is the manuscript technically sound, and do the data support the conclusions?

The manuscript must describe a technically sound piece of scientific research with data that supports the conclusions. Experiments must have been conducted rigorously, with appropriate controls, replication, and sample sizes. The conclusions must be drawn appropriately based on the data presented.

Reviewer #3: Yes

Reviewer #4: No

The two authoritative literatures recently published on underwater sound absorption of periodic structures have not been experimentally studied [1-2], and according to a 2018 paper, strongly coupled systems are more sensitive to changes in boundary conditions. Therefore, the author speculates that there may be a significant difference between experimental and theoretical results. Therefore, this literature did not conduct experiments. For non-periodic structures, sound absorption can be measured in the underwater impedance tube equipment [3].

[1] Fang, X., Yin, X., Wu, J., Li, Y., Li, H., Wang, W., Li, Y., & Wu, W. (2023). Underwater metagratings for sub-kilohertz low frequency and broadband sound absorption. International Journal of Mechanical Sciences.

[2] Hu, N., Jin, J., Peng, W., Zhang, Z., & Hu, H. (2024). Liquid-solid synergistic mechanism sound absorption for underwater anechoic coating. International Journal of Mechanical Sciences.

[3] Zhang, H., Shi, D., Zha, S., & Wang, Q. (2018). A simple first-order shear deformation theory for vibro-acoustic analysis of the laminated rectangular fluid-structure coupling system. Composite Structures.

3. Has the statistical analysis been performed appropriately and rigorously?

Reviewer #3: Yes

Reviewer #4: N/A

This article does not involve statistical analysis issues, and the relevant results are converged. It should be noted that two conditions need to be strictly met to adopt the analytical model ($\omega_{0}r/c_{l}$<<1 and $\omega_{0}a/c_{l}$<<1 where *c_l_* denotes the longitudinal speed in the PDMS) [1,2].

[1] Fang, X., Yin, X., Wu, J., Li, Y., Li, H., Wang, W., Li, Y., & Wu, W. (2023). Underwater metagratings for sub-kilohertz low frequency and broadband sound absorption. International Journal of Mechanical Sciences.

[2] G.S. Sharma, A. Skvortsov, I. MacGillivray, N. Kessissoglou (2019). Acoustic performance of periodic steel cylinders embedded in a viscoelastic medium. Journal of Sound and Vibration, 443 652-665.

4. Have the authors made all data underlying the findings in their manuscript fully available?

The PLOS Data policy requires authors to make all data underlying the findings described in their manuscript fully available without restriction, with rare exception (please refer to the Data Availability Statement in the manuscript PDF file). The data should be provided as part of the manuscript or its supporting information, or deposited to a public repository. For example, in addition to summary statistics, the data points behind means, medians and variance measures should be available. If there are restrictions on publicly sharing data—e.g. participant privacy or use of data from a third party—those must be specified.

Reviewer #3: Yes

Reviewer #4: (No Response)

The authors have made all data underlying the findings described in their manuscript fully available without restriction.

5. Is the manuscript presented in an intelligible fashion and written in standard English?

PLOS ONE does not copyedit accepted manuscripts, so the language in submitted articles must be clear, correct, and unambiguous. Any typographical or grammatical errors should be corrected at revision, so please note any specific errors here.

Reviewer #3: Yes

Reviewer #4: (No Response)

After our repeated checks, we have made every effort to improve our article. All modifications have been marked in the article.

6. Review Comments to the Author

Please use the space provided to explain your answers to the questions above. You may also include additional comments for the author, including concerns about dual publication, research ethics, or publication ethics. (Please upload your review as an attachment if it exceeds 20,000 characters)

Reviewer #3: The manuscript focuses on the broadband sound absorption of acoustic coatings with multi-layer solid inclusions, and adopts effective medium approximation and transfer matrix method to achieve theoretical prediction of sound absorption coefficients, and then optimizes the global sound absorption characteristics through genetic algorithm. Although this manuscript is complete and logical, there are still the following main issues to be improved:

1. Please highlight the innovative nature of the manuscript, both from the perspective of structural design, research methodology, and even more so, the research findings.

We gratefully thank the referee for the expert review and insightful comments.

First, acoustic coatings with periodic multi-layer solid inclusions are proposed.

Second, effective medium approximation and transfer matrix method are adopted to achieve theoretical prediction of sound absorption coefficients for acoustic coatings with multi-layer solid inclusions.

Third, instead of intensive trials on numerical simulations, the genetic algorithm and the transfer matrix method are used to achieve the broadband absorption for the two-layer case.

Fourth, the optimized structure has significantly improved its sound absorption performance within the wide frequency range of 1780 to 8890Hz via subwavelength thicknesses.

Fifth, our study found that for the two-layer case, the distance between the inclusions has great impact on the sound absorption performance.

2. The manuscript mentions that the EFFECTIVE MEDIUM APPROXIMATION has been modified, may I ask if the solution accuracy has been improved with respect to the literature [13]? Alternatively, has the applicable range of the method been extended? Please give specific comparison and explanation.

The entire article does not mention that the theory of the literature [13] has been modified, but the word "modified" is indeed mentioned in this article, for example: “In order to facilitate readers to better understand the solution ideas, the order of the formulas has been modified.”, “*m* (*n*) order modified Bessel functions.”.

Distinct from previous studies in the literature [13], the genetic algorithm and the transfer matrix method (EFFECTIVE MEDIUM APPROXIMATION) are used to achieve the broadband absorption for the two-layer case. It is worth pointing out that our personal computer (Intel(R) Core(TM) i7-8700 CPU @ 3.20GHz 3.19 GHz, RAM 16 GB) can obtain the optimized results in 4 minutes, while the joint simulation calculation using Comsol and MATLAB is very slow and basically cannot obtain the results.

3. In the last sentence of the second paragraph on page 7, Appendix A and B correspond to a description that does not correspond to the text.

We thank the referee for pointing out this mistake. The supplementary materials have been revised, Appendix A and B correspond to the description that correspond to the text.

“**Appendix A. Transfer matrix method**

After the effective density, wave number, and thickness of each layer are achieved, the surface impedance of each layer of the structure can be derived utilizing the transfer matrix method , which relates the sound pressure and velocity between the water (*P_w_*,*V_w_*) and air domain (*P_a_*,*V_a_*) is

$\left(\begin{matrix} P_{w} \\ V_{w} \end{matrix}\right)=T\left(\begin{matrix} P_{a} \\ V_{a} \end{matrix}\right)=\left(\begin{matrix} t_{11} t_{12} \\ t_{21} t_{22} \end{matrix}\right)\left(\begin{matrix} P_{a} \\ V_{a} \end{matrix}\right)$ (s1)

in which

(s2)

Here *s* = 1, 2, …, *s* represents the serial number of the transfer matrices (s = 6 in Fig. 1(b)). *k_s_* ($\omega_{s}/c_{l}$, where *c_l_* stands for longitudinal wave speed.) and *h_s_* stand for the wave number and the transmission length of the *s* th layer medium. Therefore, the reflection and transmission coefficients can be calculated by

$t_{p}=\frac{2}{t_{11}+t_{12}/Z_{tr}+Z_{in}t_{21}+t_{22}Z_{in}/Z_{tr}}$ (s3)

$r_{p}=\frac{t_{11}+t_{12}/Z_{tr}-Z_{in}t_{21}-t_{22}Z_{in}/Z_{tr}}{t_{11}+t_{12}/Z_{tr}+Z_{in}t_{21}+t_{22}Z_{in}/Z_{tr}}$ (s4)

where *Z_in_* and *Z_tr_* are the impedance of the incident (water) and transmission (air) domains, respectively. Then the absorption coefficient can be computed as

$\alpha=1-\frac{Z_{tr}}{Z_{in}}\left|t_{p}\right|^{2}-\left|r_{p}\right|^{2}$ (s5)

**Appendix B.** **Finite Element Simulation**

To verify the correctness of the proposed algorithm, a finite element simulation in the frequency domain by using the COMSOL Multiphysics is carried out. Only one unit cell is considered due to the periodicity. As shown in fig. S1, the water and air domain are imitated by the acoustic module, and the PDMS domain and steel are simulated by the solid mechanical module. Periodic boundary conditions are applied on the upper and lower boundaries, while the perfect matching layer (PML) is used to eliminate reflection on the left and right boundaries. The structural-acoustic interaction is adopted between the fluid domain and solid domain. The background pressure field (water domain) simulates the incident acoustic wave of unity pressure amplitude. The entire region is divided into triangular meshes, the size of which is controlled by the physical field. The reflected and transmitted pressures can be obtained at the interface between the fluid and solid media on the incidence and transmission sides, respectively .

Fig. S1 Simulation settings for the single-layer structure in COMSOL Multiphysics.

**Appendix C. Genetic Algorithm**

The genetic algorithm is used to determine the geometrical parameters of the multilayer structure with broadband sound absorption. The assumed total thickness of the structure is 60 mm. The parameters that are primarily optimized contain *r*_1,_ *r*_2_, *a*_1_= *a*_2_=*a*, *t*_1_, *t*_2_. The definition of the optimization vector and constraint range for these parameters is as following

$\mathop{x}\limits^{\to} =[r_{1},r_{2},a,t_{1},t_{2}]$ (s6)

where $r_{1}\in\left[1,9\right]\text{mm}$,$r_{2}\in\left[1,9\right]\text{mm}$,$a\in\left[5,60\right]\text{mm}$,$t_{1}\in\left[10,50\right]\text{mm}$,$t_{2}\in\left[10,50\right]\text{mm}$.

The determination of the objective function involves finding the maximum value of the average absorption coefficient within a designated frequency range (*f*_min_, *f*_max_), is denoted as

$\text{Max:}\left\{\alpha\right\}=\frac{\sum_{f_{\min}}^{f_{\max}} \alpha\left(f_{i},\mathop{x}\limits^{\to} \right)}{\Delta f}$ (s7)

where ∆*f*= *f*_min_-*f*_max_.

**References**

1.	Sharma GS, Skvortsov A, MacGillivray I, Kessissoglou N. Acoustic performance of gratings of cylindrical voids in a soft elastic medium with a steel backing. The Journal of the Acoustical Society of America. 2017;141:4694-704.

2.	Fang X, Yin X, Wu J, Li Y, Li H, Wang W, Li Y, Wu W. Underwater metagratings for sub-kilohertz low frequency and broadband sound absorption. International Journal of Mechanical Sciences. 2023;260:108630.

3.	Sharma GS, Skvortsov A, MacGillivray I, Kessissoglou N. Sound absorption by rubber coatings with periodic voids and hard inclusions. Applied Acoustics. 2019;143:200-10.

4.	Cao Z, Li X, Liu B. Broadband sound absorption of a hybrid absorber with shunt loudspeaker and perforated plates. Applied Acoustics. 2023;203:109185.

5.	Li X, Liu B, Chang D. An acoustic impedance structure consisting of perforated panel resonator and porous material for low-to-mid frequency sound absorption. Applied Acoustics. 2021;180:108069.

6.	Li X, Cao Z, Li Z, Liu B. Sound absorption of a shunt loudspeaker on a perforated plate. Applied Acoustics. 2022;193:108776.”

4. Following on from the previous comment, in the first sentence of the last paragraph on page 10, the corresponding Appendix C is also inconsistent with the description in the main text.

We thank the referee for pointing out this mistake. The corresponding Appendix C is also revised to be consistent with the description in the main text.

5. The description of the transfer matrix method and the finite element method in the caption of Fig. 2 is completely contrary to that in the legend.

We thank the referee for pointing out this problem. The description has been revised as follows: “Fig. 2. sound absorption spectrum calculated by transfer matrix method (dots) and finite element method (solid line). (a) one layer of hard inclusions, (b) two layers of hard inclusions, (c) three layers of hard inclusions.”.

6. The abstract needs to be further condensed, with redundant background and lack of description of results.

We thank the referee for the valuable advice. The abstract has been revised as follows: “Viscoelastic materials with periodic multilayered hard inclusions have particular practical value in the stealth of underwater equipment, as they can absorb low-frequency and broadband sound waves. In this paper, acoustic coatings containing multi-layers of periodic solid inclusions are investigated by the transfer matrix method and the finite element method, which is further optimized by the genetic algorithm for broadband sound absorption. An analysis model is first derived to calculate effective parameters of the layer of solid scatters. Instead of intensive trials on numerical simulations, the genetic algorithm and the transfer matrix method are used to achieve the broadband absorption for the two-layer case. The optimized structure has significantly improved its sound absorption performance within the wide frequency range of 1780 to 8890Hz via subwavelength thicknesses. The optimized results show that for the two-layer case, the distance between the inclusions has great impact on the sound absorption performance. Finally, the underlying mechanism of broadband sound absorption is explained. This study can provide theoretical guidance for the design and optimization of acoustic coatings with multi-layer hard inclusions.”.

Reviewer #4: The authours propose a theoretical model for calculating the sound absorption of acoustic coatings with multiple layers of solid inclusions using the transfer matrix method. The effect of geometric parameters on sound absorption is investigated. In order to obtain better sound absorption performance, the genetic algorithm is used to optimize the acoustic coating with two layers of hard inclusions. This work is not well researched and is not worthy of publication until it is thoroughly revised.

1. This article lacks key principle descriptions and literature citations to support the theoretical modelling process, and the model is unconvincing.

Key principle descriptions and literature citations that can support the theoretical modelling process are given in literature [13].

2. The details of the numerical model are not given, and the relevant numerical results are not analyzed in depth.

The numerical model is provided in the supplementary materials, and the relevant theoretical analysis has also been improved.

3. The language and figures need further improvement.

We thank the referee for the valuable suggestions. After our repeated checks, we have made every effort to improve our article. All modifications have been marked in the article. The language and figures have been improved.
