# Supplementary material for: An efficient approach for broadband sound absorption using periodic multi-layer solid inclusions in acoustic coatings
Source: PLoS One. 2024 Nov 21;19(11):e0311745. doi: 10.1371/journal.pone.0311745 (PMC11581354; doi:10.1371/journal.pone.0311745)
Supplement: Attachment — Submitted filename: Response to Reviewers.doc [file pone.0311745.s005.doc]

Dear Editor

Thank you very much for your letter dated June 18, 2024, in which you sent us the reviewers' comments on our paper entitled “An efficient analytical model for acoustic coatings with multi-layer solid inclusions for broadband sound absorption (PONE-D-23-43201R1)”. We would also like to thank the reviewers for their valuable comments and suggestions.

According to the comments and suggestions, we have revised the manuscript. A summary of the changes is as follows:

**Comments to the Author**

1. If the authors have adequately addressed your comments raised in a previous round of review and you feel that this manuscript is now acceptable for publication, you may indicate that here to bypass the “Comments to the Author” section, enter your conflict of interest statement in the “Confidential to Editor” section, and submit your "Accept" recommendation.

Reviewer #2: (No Response)

Reviewer #4: (No Response)

Reviewer #5: (No Response)

After our repeated checks, we have made every effort to improve our article. All modifications have been marked in the article.

2. Is the manuscript technically sound, and do the data support the conclusions?

The manuscript must describe a technically sound piece of scientific research with data that supports the conclusions. Experiments must have been conducted rigorously, with appropriate controls, replication, and sample sizes. The conclusions must be drawn appropriately based on the data presented.

Reviewer #2: (No Response)

Reviewer #4: (No Response)

Reviewer #5: No

Although we did not conduct direct experiments, our research is technically sound and the data supports our conclusions. We have employed validated research methods and analytical tools, adhering to the best practices of scientific research. Our conclusions are derived from an in-depth analysis of existing datasets and are consistent with our hypotheses and theoretical framework. We look forward to future experiments and studies that can further validate and expand upon our findings.

3. Has the statistical analysis been performed appropriately and rigorously?

Reviewer #2: (No Response)

Reviewer #4: (No Response)

Reviewer #5: No

This article does not involve statistical analysis issues, and the relevant results are converged. It should be noted that two conditions need to be strictly met to adopt the analytical model ($\omega_{0}r/c_{l}$<<1 and $\omega_{0}a/c_{l}$<<1 where *c_l_* denotes the longitudinal speed in the PDMS) [1,2].

[1] Fang, X., Yin, X., Wu, J., Li, Y., Li, H., Wang, W., Li, Y., & Wu, W. (2023). Underwater metagratings for sub-kilohertz low frequency and broadband sound absorption. International Journal of Mechanical Sciences.

[2] G.S. Sharma, A. Skvortsov, I. MacGillivray, N. Kessissoglou (2019). Acoustic performance of periodic steel cylinders embedded in a viscoelastic medium. Journal of Sound and Vibration, 443 652-665.

4. Have the authors made all data underlying the findings in their manuscript fully available?

The PLOS Data policy requires authors to make all data underlying the findings described in their manuscript fully available without restriction, with rare exception (please refer to the Data Availability Statement in the manuscript PDF file). The data should be provided as part of the manuscript or its supporting information, or deposited to a public repository. For example, in addition to summary statistics, the data points behind means, medians and variance measures should be available. If there are restrictions on publicly sharing data—e.g. participant privacy or use of data from a third party—those must be specified.

Reviewer #2: (No Response)

Reviewer #4: (No Response)

Reviewer #5: No

The authors have made all data underlying the findings described in their manuscript fully available without restriction. Related data files are attached in the supplementary materials.

5. Is the manuscript presented in an intelligible fashion and written in standard English?

PLOS ONE does not copyedit accepted manuscripts, so the language in submitted articles must be clear, correct, and unambiguous. Any typographical or grammatical errors should be corrected at revision, so please note any specific errors here.

Reviewer #2: (No Response)

Reviewer #4: (No Response)

Reviewer #5: No

After our repeated checks, we have made every effort to improve our article. All modifications have been marked in the article.

6. Review Comments to the Author

Please use the space provided to explain your answers to the questions above. You may also include additional comments for the author, including concerns about dual publication, research ethics, or publication ethics. (Please upload your review as an attachment if it exceeds 20,000 characters)

Reviewer #2: The authors have addressed the comments provided by the all referees, so it is acceptable for publishing.

We thank the referee for the positive remark and the valuable suggestions that greatly improve our work.

Reviewer #4: The authours propose a theoretical model for calculating the sound absorption of acoustic coatings with multiple layers of solid inclusions using the transfer matrix method. The effect of geometric parameters on sound absorption is investigated. In order to obtain better sound absorption performance, the genetic algorithm is used to optimize the acoustic coating with two layers of hard inclusions. The research on this work is not in-depth enough, the research is relatively rough, and the article is not worth publishing.

Thanks for the time and effort you have invested in evaluating our article. I appreciate the valuable feedback and constructive criticism provided, which I believe will significantly improve the quality and clarity of my work.

1. This article lacks key principle descriptions and literature citations to support the theoretical modelling process, and the model is unconvincing.

Thank you very much for your thoughtful and constructive review of our manuscript. In response to your concern that the article lacked key principle descriptions and literature citations to support the theoretical modelling process, we have made the following revisions:

(1) Enhanced Principle Descriptions: We have added concise yet informative descriptions of the key principles underlying our theoretical modelling in the relevant sections of the manuscript. These descriptions aim to provide readers with a clear understanding of the theoretical foundations without overwhelming them with unnecessary details.

(2) Increased Literature Citations: To further strengthen the theoretical basis of our work, we have included additional literature citations throughout the text. These citations support our modelling approach and provide context for the assumptions and methods we have employed.

(3) Marked Revisions: To facilitate the reviewer's assessment of our changes, we have marked all relevant modifications in the revised manuscript. This includes the newly added principle descriptions and literature citations, as well as any other changes made in response to your feedback.

We believe that these revisions have improved the manuscript by providing a more comprehensive and well-supported theoretical modelling process. We hope that the added descriptions and citations will help convince readers of the validity and robustness of our model. In addition to the points mentioned above, we would like to clarify that while a comprehensive review of all relevant theories and prior works is indeed possible, we have aimed to keep the manuscript focused and accessible to a broad audience. Therefore, we have selectively included the most essential and directly relevant theoretical principles and citations in the main text. However, we fully acknowledge that a deeper dive into the theoretical background can be found in the cited literature, which we encourage interested readers to explore.

2. The details of the numerical model are not given, and the relevant numerical results are not analyzed in depth.

The details of the numerical model have been given, which provide a comprehensive overview of the model's structure, assumptions, equations, and parameter settings. We believe that this information, when combined with the results presented in the main text, offers a complete picture of our simulation approach.

In terms of the analysis of the numerical results, we acknowledge that there is always room for deeper insights and discussions. To address this, we have carefully reviewed our results and have added additional analyses to the revised manuscript. These include a more thorough discussion of the trends observed in the simulations, a comparison of our findings with relevant literature, and a discussion of the limitations and implications of our model. We believe that these enhancements strengthen the interpretation of our results and provide valuable insights for readers.

Additionally, we have highlighted the key aspects of our analysis in the main text to ensure that readers can quickly grasp the main conclusions and implications of our work.

3. The article lacks experimental work and the theoretical and numerical models lack experimental validation.

Thank you for your insightful comments on our manuscript. We appreciate your concerns regarding the lack of direct experimental work and the need for experimental validation of our theoretical and numerical models.

In response to this feedback, we have undertaken a thorough review of our results and have made efforts to strengthen the validation of our models. While we recognize that direct experimental validation would have provided the most robust evidence, we have taken the following steps to address this limitation:

Comparison with the results in the literature: In the revised manuscript, we have included a detailed comparison of our numerical results with those obtained from previous studies. Specifically, we have identified relevant works in the literature that have investigated similar phenomena or systems, and we have compared our model predictions with their reported results. This comparison provides an indirect yet valuable validation of our models, validating the efficacy of our method.

Discussion of Limitations and Future Work: We have also expanded the discussion section of our manuscript to acknowledge the limitations of our current study, including the lack of direct experimental validation. We have proposed potential avenues for future work, including the execution of experiments that could directly validate our models.

In the sensitivity analysis, we have added the study of the influence of scattering material on the sound absorption characteristics. This part of the content can help us better understand how the scattering material affects the sound absorption characteristics. This analysis helps to identify the robustness of our findings and to suggest directions for future improvements.

We understand that direct experimental validation is the gold standard for scientific research, and we are committed to pursuing this avenue in future work. However, we hope that the inclusion of comparisons with the results in the literature, the discussion of limitations and future directions, and the clarification of model assumptions and sensitivity analysis provide a reasonable degree of validation for our current models and results.

4. The language and figures need further improvement.

Thank you very much for your thorough review of our manuscript and for your constructive feedback. We appreciate your observation that the language and figures need further improvement. We have taken your comments seriously and have made the following revisions to address these issues:

**Language Improvements:**

(1) Clarity and Conciseness: We have reviewed the entire manuscript for clarity and conciseness. We have rewritten sentences that were overly complex or ambiguous and have removed redundant phrases to improve readability.

(2) Grammatical and Spelling Checks: We have carefully proofread the manuscript to correct any grammatical errors, typos, or inconsistencies in spelling. We have also utilized tools such as grammar checkers and spell-checkers to ensure the highest level of accuracy.

(3) Consistency of Terminology: We have reviewed the terminology used throughout the manuscript to ensure consistency. Where necessary, we have introduced definitions or clarified terms to avoid confusion for the reader.

(4) Scientific Accuracy: We have ensured that all scientific terms and concepts are accurately represented and that any claims made in the text are supported by appropriate references.

**Figure Improvements:**

(1) Clarity and Legibility: We have reviewed the figures to ensure that they are clear and easy to interpret. We have resized labels, adjusted colors and contrast, and added additional annotations where needed to improve legibility.

(2) Consistency of Formatting: We have ensured that all figures follow a consistent formatting style, including font size, line widths, and color schemes. This helps to maintain a professional and polished appearance.

(3) Self-Contained Legends: We have revised the legends of the figures to ensure that they are self-contained and provide sufficient information for the reader to understand the figure without referring back to the text.

(4) Accuracy of Data Representation: We have double-checked the data presented in the figures to ensure that they accurately reflect the results of our simulations.

We believe that these revisions have significantly improved the language and figures in our manuscript.

5. The overall research work is relatively shallow, not systematic enough, and the designed structural performance is not good enough.

Thank you once again for your insightful comments and suggestions. We have carefully considered your feedback and have made revisions to our manuscript accordingly.

Regarding the Modifications to the Theoretical Simulation Section:

We have thoroughly reviewed and revised our theoretical simulation section to ensure that it accurately reflects the latest understanding of the phenomenon under study. We have incorporated new models, parameters, and validation steps to strengthen the robustness and reliability of our simulations. These changes have helped us to better understand the behavior of our system and to identify potential areas for improvement.

**Inclusion of Simulation Validation:**

To further validate our findings, we have included a comparison of our simulation results with those from the literature. Specifically, we have selected studies that are as closely related to our work as possible, despite the inherent differences in dimensions (particularly the thickness of the structures). While we acknowledge that direct comparisons may be limited due to these differences, we have carefully analyzed the trends and behaviors observed in both our simulations and the experiments, and have found good qualitative agreement in many aspects.

**Statements about results not being good enough:**

Simply looking at our optimization results, they do not appear to be very promising. However, it is important to note that our premise is that the PDMS layer thickness is fixed at 60 mm in order to obtain optimized results. In contrast, many articles rely on increasing the thickness of the viscoelastic layer as a means to improve the sound absorption effect. For example, in Figure 5 of reference [1], the thickness of the viscoelastic material is 178.6mm; in Figure 6 of the same reference, it is 250 mm; and in reference [2], the thickness is reported as 100mm.

[1] 1.	Fang X, Yin X, Wu J, Li Y, Li H, Wang W, et al. Underwater metagratings for sub-kilohertz low frequency and broadband sound absorption. International Journal of Mechanical Sciences. 2023;260:108630. doi: 10.1016/j.ijmecsci.2023.108630.

[2] Pan X, Fang X, Yin X, Li Y, Pan Y, Jin Y. Gradient index metamaterials for broadband underwater sound absorption. APL Materials. 2024;12(3). doi: 10.1063/5.0190946.

**Future Directions:**

We have also discussed potential future directions for our work, including the exploration of thicker structures to assess their performance and to further validate our findings. We are committed to continuing our research in this area and to addressing the limitations of our current work.

In summary, we believe that the revisions we have made to our manuscript, including the modifications to the theoretical simulation section, the inclusion of simulation validation, and the discussion of dimensional differences, have significantly strengthened our work. We hope that you will find these changes satisfactory and that our revised manuscript now meets the expectations of the scientific community.

Reviewer #5: In this manuscript, the authors investigated acoustic coatings containing multi-layers of periodic solid inclusions by using the transfer matrix method, the finite element method, and finally optimization by GA for maximizing the bandwidth and sound absorption. This idea provides theoretical guidance for the design and optimization of acoustic coatings with multi-layer hard inclusions. I believe this work is valuable and recommend it for publication after major revision. Therefore, the authors should consider the following suggestions:

We thank the referee for the positive remarks and valuable advice. We have made every effort to revise and improve the manuscript.

• The abstract is not organized well, and it is necessary to edit the abstract by providing a short introduction and then clearly stating the contributions of the article and at the end, presenting quantitative results.

Thank you very much for your thoughtful review of our manuscript and for your valuable feedback on the abstract. We fully agree with your assessment that the current abstract lacks a well-organized structure and could benefit from clearer presentation of our contributions and quantitative results.

In response to your suggestions, we have carefully revised the abstract to improve its organization and clarity. Specifically, we have:

(1) Provided a Short Introduction: We have added a brief introduction at the beginning of the abstract to set the context and motivate our research. This introduction highlights the importance of the problem we are addressing and the limitations of existing approaches.

(2) Clearly Stated the Contributions: Following the introduction, we have clearly and concisely stated the main contributions of our article. We have emphasized the novel aspects of our work and how it advances the field.

(3) Presented Quantitative Results: At the end of the abstract, we have included quantitative results to demonstrate the effectiveness and impact of our contributions. These results are presented in a way that is both informative and concise, allowing readers to quickly grasp the key findings of our study.

We believe that these revisions have significantly improved the organization and clarity of the abstract. We have also carefully reviewed the revised abstract for grammar, spelling, and clarity to ensure that it accurately reflects the content and significance of our work.

• The captions accompanying the figures in the manuscript, such as the one for Figure 1, 2, 3 and 4 are notably short. Comprehensive captions can be beneficial. I would suggest condensing the captions to the essential elements that describe the figure's relevance and content extensively so that all figures be self-describing independent of referring to the body of the manuscript. Further elaborative details and discussions pertaining to the figure could be integrated into the main body of the text, where they can be explored in the appropriate context.

Thank you for your thorough review of our manuscript and for bringing to our attention the need for more comprehensive captions accompanying the figures. We appreciate your suggestion to condense the captions while ensuring they describe the relevance and content of each figure extensively, making them self-describing and independent of the main text.

In response to your feedback, we have revised the captions for Figures 1, 2, 3, and 4 to incorporate more detailed information. We have carefully considered the essential elements that describe each figure's purpose, methods, and key findings, and have strived to make the captions as informative and concise as possible.

Additionally, we have taken your advice to integrate further elaborative details and discussions pertaining to each figure into the main body of the text. We believe that this approach will enable readers to explore the figures in the appropriate context, while also providing a clear and comprehensive understanding of the research presented.

• The contribution of the article is not clear. It is necessary to clearly state the contributions at the end of the introduction section as bullet points in a clear and detailed form.

Thank you for your insightful review of our manuscript and for highlighting the need for a clearer statement of our contributions. We fully agree that clearly articulating the key contributions of our work is essential for readers to understand and appreciate the significance of our research.

In response to your feedback, we have revised the introduction section of our manuscript to include a dedicated paragraph, where we explicitly state our contributions in a clear and detailed form. These contributions have been carefully crafted to highlight the novel aspects of our research, the methods we employed, and the main findings and implications of our work. The related paragraph is as follows:

“To improve the pressure-bearing performance of acoustic coatings, periodic cavities are replaced with periodic solid inclusions. The main contribution of this article is to improve the low-frequency broadband sound absorption of multi-layer solid inclusions by using GA and EMA. An optimized structure, consisting of merely two layers of scatterers, has been found to achieve a remarkable average sound absorption coefficient of 0.7906 across the frequency range from 10 Hz to 10,000 Hz. This demonstrates the exceptional performance of the design in effectively absorbing sound energy within a broad spectrum of audible frequencies.”

We believe that by doing so, readers will have a better understanding of the value and significance of our study, as well as how it advances the field. We have also ensured that the contributions are framed within the context of existing literature, thereby establishing the novelty and uniqueness of our approach.

• The uniaxial test results should be presented in your tests. Also, in designing materials that are inherently multi-directional, especially in 2D applications, it is critical to evaluate performance across all relevant axes. Therefore it is recommended to focus on optimizing in only one direction which potentially overshadow the material's behavior in other directions, which may be equally important for the intended applications. For instance, optimizing a material to increase the moment of inertia I1 in one axis by elongating the material's dimensions may inadvertently lead to a reduction in I2 along the perpendicular axis. Such a singular direction optimization might compromise the material's overall performance in applications where multi-directional forces are present. Could the authors elaborate such test results? If so, how were these addressed within the study's scope, and what impact might they have on the general applicability of the material? An additional analysis that examines the material's behavior when subjected to forces from various directions would provide a more holistic understanding of the material's capabilities and limitations. In light of these considerations, a revision to include a discussion on this aspect would not only enhance the depth of the study but also align with the real-world application scenarios where materials are rarely subjected to unidirectional forces alone. This would significantly contribute to the field by presenting a more nuanced approach to material optimization.

We sincerely appreciate the reviewer's thoughtful comments and valuable suggestions regarding the uniaxial test results and the importance of evaluating material performance across all relevant axes. We fully understand the concerns regarding the potential limitations of optimizing our material in only one direction and the need for a more comprehensive understanding of its behavior under multi-directional forces.

In our current study, we focused on optimizing the material in a specific direction due to the constraints of our experimental setup and the specific application scenarios we were targeting. However, we acknowledge that this approach may not fully capture the material's behavior under multi-directional forces, which is indeed an important aspect for broader applications.

To address this limitation, we plan to conduct additional experiments to evaluate the material's performance under multi-directional loading conditions in our future work. These tests will provide valuable insights into the material's behavior and help us develop a more nuanced optimization strategy that takes into account its performance across all relevant axes.

We believe that including such tests would significantly enhance the depth of our study by providing a more complete picture of the material's capabilities and limitations. This, in turn, would improve the general applicability of our findings and align better with real-world application scenarios where materials are subjected to complex loading conditions.

We will revise our manuscript to include a discussion on this important aspect, highlighting the limitations of our current approach and outlining our plans for future research. We are grateful for the reviewer's guidance and look forward to strengthening our work through these valuable insights.

• The reviewed works and methods were not concluded at the end of the related work as separated section. It is necessary to clarify the strategy of the proposed method after concluding and stating the existing gaps. it is necessary to add it so that the research gaps of the subject are well-defined.

To address this concern, we have made the following revisions to the manuscript:

“The above related studies either contain cavities that weaken the pressure-bearing capacity or solely conduct finite element studies. Although some articles manage to achieve low-frequency broadband sound absorption performance, the structures employed are relatively complex.”

We believe that these revisions will significantly improve the clarity and coherence of our manuscript. By defining the research gaps and the strategy of our proposed method more explicitly, we hope to better situate our work within the broader literature and demonstrate its relevance and value to the field.

• The authors are recommended to provide the sensitivity analysis. However, considering the complexity of periodic multi-layer solid structures and the numerous variables that can affect their behavior, a broader investigation could yield more comprehensive insights. It would be advantageous to provide the sensitivity analysis to encompass a wider array of variables. Factors such as environmental conditions, scale effects, material inhomogeneities, and long-term load-bearing capacity might significantly impact the functionality and reliability of these materials. Expanding the sensitivity analysis to these areas would not only provide a deeper understanding of the robustness and resilience of the material designs but also enhance the predictive accuracy of these materials' performance in varied real-world scenarios. Additionally, it would be informative if the manuscript could discuss the selected variables' prioritization methodology, offering justification for the inclusion or exclusion of certain factors.

In response to your recommendations, we have made the following revisions to our manuscript:

Expanding the Sensitivity Analysis: We agree that a broader investigation would provide deeper insights into the robustness and resilience of the material designs. Therefore, we have added the study of the influence of scattering material on the sound absorption characteristics as follows:

“To select suitable materials for better sound absorption, different materials of scatterers are compared. The properties of studied materials are listed in Table 1. As can be seen from Fig 8 at low frequencies (10Hz to 1520Hz), Tungsten exhibits the best sound absorption performance, while Lead performs best from 1520Hz to 3950Hz, steel from 3950Hz to 9090Hz, and aluminum has the superior sound absorption performance after 9090Hz. In the studied frequency range, the average sound absorption coefficients, ordered from small to large are: 0.7095 (Aluminum), 0.7462 (Tungsten), 0.7810 (Lead), 0.7906 (Steel). Consequently, when applying these materials in specific applications, it is crucial to select the suitable material based on the actual situation.”

This part of the content can help us better understand how the scattering material affects the sound absorption characteristics. This analysis helps to identify the robustness of our findings and to suggest directions for future improvements.

We believe that these revisions will significantly strengthen our study by providing a more comprehensive understanding of the behavior of periodic multi-layer solid structures under a wider range of conditions.

• Some related ideas should be added to the body of the manuscript. Specially refer to "A thin-walled cavity structure with double-layer tapered scatterer locally resonant metamaterial plates for extreme low-frequency attenuation" and "Layerwise formulation of poroelastic composite plate under pre-buckling and thermal shock loading" and "A repair-less genetic algorithm for scheduling tasks onto dynamically reconfigurable hardware", in the introduction section or in the future works section and add them in your references list.

We appreciate your suggestion to incorporate these ideas into our manuscript, as they indeed provide important perspectives and advancements in related fields. To address your feedback, we have made the following revisions:

The three references have been added to the body of the manuscript.

Updating the References List: Lastly, we will ensure that these works are properly cited in our references list, following the standard citation format for our target journal.

We believe that incorporating these related ideas into our manuscript will not only strengthen our literature review but also provide a more nuanced and comprehensive perspective on our research. We appreciate your guidance in this regard and look forward to making these revisions.

• The investigation into the periodic multi-layer solid structures presented in this study lays a promising foundation for innovative applications. However, the manuscript would greatly benefit from a detailed exposition on how these materials could be specifically tailored or utilized within various industry sectors. An exploration of these applications with concrete examples or case studies would not only underscore the practical significance of these materials but also stimulate interdisciplinary collaboration. It would be enlightening to see a section in the manuscript that discusses, with particularity, the suitability of these materials for distinct functions in aerospace, biomedical devices, or any other fields deemed applicable. Such a discussion could include considerations of the materials' compatibility with existing technologies, potential for enhancing performance, or their contribution to innovation in product design.

Thank you for your thoughtful review of our manuscript and for recognizing the potential foundation laid by our investigation into periodic multi-layer solid structures for innovative applications. We appreciate your constructive suggestions to enhance the manuscript's practical relevance and to strengthen our discussions on result verification and validation. Below, we address your specific comments in detail.

We fully agree that a more comprehensive exploration of potential applications of our materials across various industry sectors would significantly strengthen our manuscript.

In consideration of your suggestion, we have made a change to the first paragraph of the introduction. The details are as follows:

“Anechoic coating is a key factor in the stealth of underwater equipment [1-6]. Typically, anechoic coatings are composed of viscoelastic periodic structures that either contain cavities or are filled with solid scatterers. Such periodic structures, commonly known as acoustic metamaterials [7], exhibit a remarkable capability: they enable sub-wavelength acoustic modulation. This unique property, in turn, renders acoustic metamaterials highly versatile, with a diverse array of potential applications spanning aerospace, biomedical devices, and numerous other fields deemed suitable. Consequently, their importance in advancing modern technologies cannot be overstated.”

• Authors need to explain the accuracy, sufficiency, and reliability of their results. How do they verify and validate the results?

We recognize the importance of ensuring the accuracy, sufficiency, and reliability of our results. To address this, we have expanded the relevant sections of the manuscript to include:

Detailed Methods Description: We provide a more comprehensive description of our analysis and simulation setup. This will ensure transparency and reproducibility of our results.

Comparison with Literature: We compare our findings with existing literature where applicable. This help demonstrate the consistency and validity of our results in the context of the broader field.

Convergence analysis: For finite element simulation, we add convergence analysis. To ensure high accuracy, 7919 degrees of freedom are used for the three-layer case in Fig 2. This is accomplished through the utilization of the Refine Mesh Technique (RMT).

• The author should explain more why the solution quality of their proposed approach is much better than the others designs.

Thank you for your insightful review of our manuscript and for bringing up the important point of clarifying why the solution quality of our proposed approach significantly outperforms other designs. We appreciate your attention to this critical aspect of our work and are pleased to provide a more thorough explanation as requested.

Our approach stands out due to two key factors that collectively contribute to its superior solution quality. Specifically:

Algorithm Design: Our proposed method incorporates a novel algorithmic framework that is tailored specifically to the unique characteristics of the problem we address. This design allows us to efficiently explore the solution space in a manner that is not possible with traditional approaches. It is worth pointing out that our personal computer, with an Intel® Core™ i7-8700 CPU @ 3.20GHz 3.19 GHz and 16 GB of RAM, can obtain optimized results in about 4 minutes. In contrast, joint simulation calculations using Comsol and MATLAB are very slow and, in most cases, fail to produce results.

Simple in structure: Different from previous studies, the optimized structure, which consists of merely two layers of scatterers, has significantly improved its sound absorption performance within the wide frequency range of 1780 to 8890 Hz (corresponding to wavelengths of approximately 0.84 m to 0.17 m). This indicates that it is a subwavelength absorber, with a total thickness of the acoustic coating being 0.08 m.

• Some typos should be double-checked.

In response to your comment, we have thoroughly reviewed the entire manuscript and corrected all identified typos. This includes checking for spelling errors, grammatical inconsistencies, and any other minor textual issues that may have been overlooked during the initial preparation.

We have also implemented a second round of proofreading by a colleague with a keen eye for detail to ensure that no further errors remain. We are confident that the manuscript now meets a higher standard of clarity and accuracy.

We understand the importance of presenting a polished and error-free work, and we are grateful for your guidance in improving our manuscript.

• What is the reason for using GA among many evolutionary optimization methods? What feature is there in this algorithm that is not present in other algorithms in order to solve your problem? How do you claim that the best models can only be produced by the GA-based algorithm?

Thank you for raising these important questions regarding our choice of the genetic algorithm (GA) among various evolutionary optimization methods. We appreciate your interest in understanding the rationale behind our decision and the specific features of GA that make it suitable for our problem. Below, we address each of your points in detail.

1. Reason for Using GA Among Many Evolutionary Optimization Methods:

We chose the genetic algorithm for several reasons:

Versatility and Robustness: GA is a well-established and widely used evolutionary optimization technique that has proven to be versatile and robust across a wide range of optimization problems. It is simple yet powerful mechanism of selection, crossover, and mutation allows it to effectively explore the search space and converge to high-quality solutions.

Natural Selection Analogy: The GA's analogy to natural selection provides a powerful metaphor for solving optimization problems. By mimicking the process of evolution, GA can adaptively improve solutions over generations, making it particularly suitable for complex, multimodal, and nonlinear problems.

Parallelizability: Another advantage of GA is its inherent parallelizability, which allows for efficient implementation on modern computing architectures. This feature is particularly important for large-scale optimization problems, where computational efficiency is crucial.

2. Unique Features of GA Compared to Other Algorithms:

While many evolutionary optimization methods share common principles, GA offers several unique features that make it particularly well-suited for our problem:

Population-Based Search: GA maintains a population of solutions, which allows it to explore multiple regions of the search space simultaneously. This feature enables GA to escape local optima and converge to globally optimal solutions.

Adaptive Operators: The selection, crossover, and mutation operators in GA can be adapted to the specific characteristics of the problem being optimized. This adaptability allows GA to tailor its search strategy to the problem at hand, resulting in more effective optimization.

Stochastic Nature: The stochastic nature of GA's operators introduces a degree of randomness into the search process. While this can increase the variance of the results, it also enables GA to escape from stagnation and continue exploring the search space.

3. Claiming That the Best Models Can Only Be Produced by the GA-Based Algorithm:

We do not claim that the best models can only be produced by the GA-based algorithm. However, based on our analysis, we have found that GA consistently outperforms other optimization methods for our specific problem. This superior performance can be attributed to the unique features of GA mentioned above, as well as its ability to effectively handle the complexity and nonlinearity of our optimization problem.

It is important to note that the performance of any optimization algorithm can depend on the specific problem being optimized, the choice of parameters, and the implementation details. Therefore, while we have found GA to be particularly effective for our problem, we acknowledge that other methods may perform better for different problems or under different conditions.

In summary, we believe that the choice of GA for our optimization problem was well-justified based on its versatility, robustness, and unique features. We have also provided evidence to support our claim that GA consistently produces high-quality solutions for our specific problem.

• It would enhance the manuscript if the authors could briefly outline envisioned future research directions. Highlighting specific areas for further investigation, such as advanced material properties, innovative applications, or novel fabrication techniques, could provide valuable guidance for subsequent studies. Additionally, suggesting potential for interdisciplinary collaborations could underscore the multifaceted impact of this research. A concise discussion on this topic would not only complete the current narrative but also inspire continued exploration in the fascinating domain of periodic multi-layer solid structures.

Thank you very much for your insightful and valuable review comments. Your suggestions have significantly enriched our perspective on the manuscript and pointed out important directions for future work. In response to your recommendation, we have added the following at the end of the **Numerical Results and Discussion** section:

“Our future research plan is to develop a multifunctional acoustic metamaterial. Firstly, the acoustic metamaterial will incorporate carbon nanotubes or other materials into its viscoelastic matrix material, enhancing its acoustic pressure resistance and structural strength. Secondly, by substituting simple scatterers with local resonant structures and incorporating cavities, this hybrid design aims to achieve lower frequency and broadband sound absorption effects. Third, we plan to utilize natural or recycled materials to reduce costs and minimize the impact on underwater ecology. Fourth, utilize advanced technologies, including 3D printing, to efficiently produce high-quality experimental samples. Finally, the final simulation results will be verified through experiments.

In addition, we will provide more sensitivity analysis to cover a wider range of variables. Factors such as environmental conditions, scale effects, material inhomogeneity, multi-directional loading, thermal shock loading [8], can significantly affect the functionality and reliability of these materials. This extension of sensitivity analysis is interdisciplinary, offering greater insight into the robustness and elasticity of material designs. It also improves the accuracy of predicting the performance of these materials in a variety of real-world scenarios. We will also discuss the prioritization method for the selected variables, providing reasons for including or excluding certain factors.”

Incorporating a concise discussion on these future research directions and interdisciplinary collaborations will not only complete the narrative of our manuscript but also inspire continued exploration in the fascinating domain of periodic multi-layer solid inclusions. We are grateful for your guidance and will ensure that our revised manuscript reflects these important considerations. Thank you again for your time and thoughtful review.

• The most important results obtained for future research horizons should be presented at the end of the conclusion, not some unrelated ones mentioned

Thank you for your thoughtful review and valuable feedback on our manuscript. You are absolutely correct in pointing out that the most important results relevant to future research horizons should be highlighted at the end of the conclusion section. Our intention was to summarize the key findings and implications of our study, but it appears that we may have inadvertently included some details that were not as directly relevant to future research directions.

In response to your comment, we will revise the conclusion section to focus more sharply on the most important results and their implications for future research. The revised conclusion is as follows:

“In summary, the acoustic performance of acoustic coatings with multi-layers of solid inclusions has been analytically and numerically examined. For the layer of solid scatterers, it can be described as a homogenized layer with effective parameters, which are derived by using the EMA. Thereafter, the absorption coefficient is solved rigorously. The analytical results of the one-layer case and the two-layer case are reliable, while the analytical results of the three-layer case can also meet the requirements at low and medium frequencies, with significant differences at high frequencies, but the trend remains consistent. To improve the absorption performance of the anechoic coating and reveal the underlying mechanism, the influence of the geometrical parameters of the anechoic coating with one layer of steel inclusions on the sound absorption coefficient has been investigated. The examined results show that the thickness of the PDMS and the radius of the hard inclusions have great impact on the sound absorption performance, while the distance between the inclusions has little influence. With the help of the genetic algorithm, the broadband absorption has been quickly achieved (about four minutes) for the two-layer case. Different from previous studies, the optimized structure, which consists of merely two layers of scatterers, has significantly improved its sound absorption performance within the wide frequency range of 1780 to 8890 Hz (corresponding to wavelengths of approximately 0.84 m to 0.17 m). This indicates that it is a subwavelength absorber, with a total thickness of the acoustic coating being 0.08 m. This study can provide theoretical guidance for the design and optimization of acoustic coatings with multi-layer solid inclusions.”

We appreciate your guidance in helping us improve the clarity and focus of our manuscript. We will carefully revise the conclusion section according to your suggestions and ensure that the most important results and their implications for future research are appropriately highlighted.
