# Supplementary material for: An efficient approach for broadband sound absorption using periodic multi-layer solid inclusions in acoustic coatings
Source: PLoS One. 2024 Nov 21;19(11):e0311745. doi: 10.1371/journal.pone.0311745 (PMC11581354; doi:10.1371/journal.pone.0311745)
Supplement: Attachment — Submitted filename: Response to Reviewers.docx [file pone.0311745.s006.docx]

Dear Editor

Thank you very much for your letter dated August 27, 2024, in which you sent us the reviewers' comments on our paper entitled “Acoustic coatings with periodic multi-layer solid inclusions for broadband sound absorption (PONE-D-23-43201R3)”. We would also like to thank the reviewers for their valuable comments and suggestions.

According to the comments and suggestions, we have revised the manuscript. A summary of the changes is as follows:

**Comments to the Author**

1. If the authors have adequately addressed your comments raised in a previous round of review and you feel that this manuscript is now acceptable for publication, you may indicate that here to bypass the “Comments to the Author” section, enter your conflict of interest statement in the “Confidential to Editor” section, and submit your "Accept" recommendation.

Reviewer #1: (No Response)

Reviewer #5: All comments have been addressed

After our repeated checks, we have made every effort to improve our article. All modifications have been marked in the article.

2. Is the manuscript technically sound, and do the data support the conclusions?

The manuscript must describe a technically sound piece of scientific research with data that supports the conclusions. Experiments must have been conducted rigorously, with appropriate controls, replication, and sample sizes. The conclusions must be drawn appropriately based on the data presented.

Reviewer #1: Yes

Reviewer #5: Yes

3. Has the statistical analysis been performed appropriately and rigorously?

Reviewer #1: I Don't Know

Reviewer #5: Yes

This article does not involve statistical analysis issues, and the relevant results are converged. In order to ensure mesh convergence without redundancy, the Refine Mesh Technique (RMT) is used as illustrated in Fig 3. To validate the predictive model of acoustic coatings, we compare the absorption coefficients derived from the current simulation method (FEM) with those obtained by Sharma et al. [13]. As shown in Fig 4, comparing our results (blue lines) with those results (dotted lines) from reference [13], we can see that our simulated results agree reasonably with those of the reference over the entire frequency region, thus validating the present model. In addition, to validate the analytical method (TMM) utilized in this paper, results from the FEM are also presented in Fig 5 for comparison. By the above methods, we ensure that our results are credible.

4. Have the authors made all data underlying the findings in their manuscript fully available?

The PLOS Data policy requires authors to make all data underlying the findings described in their manuscript fully available without restriction, with rare exception (please refer to the Data Availability Statement in the manuscript PDF file). The data should be provided as part of the manuscript or its supporting information, or deposited to a public repository. For example, in addition to summary statistics, the data points behind means, medians and variance measures should be available. If there are restrictions on publicly sharing data—e.g. participant privacy or use of data from a third party—those must be specified.

Reviewer #1: Yes

Reviewer #5: Yes

5. Is the manuscript presented in an intelligible fashion and written in standard English?

PLOS ONE does not copyedit accepted manuscripts, so the language in submitted articles must be clear, correct, and unambiguous. Any typographical or grammatical errors should be corrected at revision, so please note any specific errors here.

Reviewer #1: Yes

Reviewer #5: Yes

6. Review Comments to the Author

Please use the space provided to explain your answers to the questions above. You may also include additional comments for the author, including concerns about dual publication, research ethics, or publication ethics. (Please upload your review as an attachment if it exceeds 20,000 characters)

Reviewer #1: This manuscript discusses research on anechoic coatings, which are crucial for enhancing the acoustic stealth of underwater equipment. The study focuses on the development and analysis of acoustic coatings containing periodic multi-layer solid inclusions to achieve broadband sound absorption. While the manuscript demonstrates innovation in both theory and methodology, there is still room for improvement in the presentation and discussion. Therefore, the publication can be considered after the authors address the following comments properly.

Thank you very much for your insightful feedback on our manuscript. We appreciate the time you’ve taken to review our work and your valuable comments. We are pleased to hear that you found our research innovative in theory and methodology. We acknowledge your observations regarding the presentation and discussion and are committed to addressing them comprehensively.

1. There are several instances in the manuscript where the language is not fluent, which affects the overall readability. I recommend that the authors perform a thorough language and grammar check of the entire manuscript to ensure clarity and readability.

Thank you for your detailed review and constructive feedback. We appreciate your observations regarding the language and readability of our manuscript.

We acknowledge that the clarity and fluency of the text are crucial for effective communication of our research findings. In response to your comments, we have performed a thorough language and grammar check of the entire manuscript. This involves:

Editing for Fluency: We have revised sections where the language may be awkward or unclear to enhance overall readability and ensure that the text flows smoothly.

Grammar and Syntax Check: We have corrected any grammatical errors and improve sentence structure to adhere to proper academic writing standards.

Proofreading: We have conducted a final proofread to ensure that the manuscript is free from typographical errors and inconsistencies.

We have performed a thorough language and grammar check of the entire manuscript, and the revised parts are all highlighted.

We believe these revisions will significantly improve the manuscript’s clarity and readability.

2. The discussion of the pros and cons of the current theoretical model, especially the transfer matrix method (TMM), is insufficient. In the methods section, it would be beneficial for the authors to clearly state the prerequisites and limitations required for the model’s calculations. Further discussion on the applicability, accuracy, and limitations of the model under different material and structural conditions is crucial for understanding its reliability and suitability.

Thank you for your insightful comments regarding the discussion of the pros and cons of our current theoretical model, particularly the transfer matrix method (TMM). We appreciate your highlighting the importance of a thorough examination of the prerequisites, limitations, applicability, accuracy, and limitations of the model under various conditions.

In response to your feedback, we have made the following revisions to our manuscript:

In the introduction section, we have added these sentences as follows: “In these studies, the transfer matrix method (TMM) played a pivotal role in modeling and analyzing the sound absorption characteristics of these complex structures. The TMM is a fast and simple method that is applicable in various physics fields, such as acoustics, quantum mechanics, and optics, to analyze wave propagation in one-dimensional systems [17, 18].” Here, we emphasize the importance of the transfer matrix method in solving the sound absorption characteristics of the complex structures, and point out that it is a fast and simple method. Moreover, it is used in multiple physical fields, indicating that it is a relatively reliable method. However, it is also pointed out that it is suitable for solving one-dimensional problems, which is also a drawback of this method.

Prerequisites and Limitations: We have clearly stated the prerequisites and limitations required for the model's calculations in the methods section as follows: “It should be noted that certain conditions must be met to apply this analytical method. First, the density of the hard inclusions must be significantly higher than that of the surrounding elastic medium [41], as Eq. (1) is derived by treating the scatterer as a rigid body. Second, the longitudinal sound speed of the host elastic medium should be much greater than its shear wave speed. Third, the analytical model used in this study is suitable for sound wavelengths that are significantly larger than both the inclusion radius *r* and the spacing *a*. In our research, the scatterer's radius is always smaller than the lattice constant, ensuring that the medium can be considered homogeneous, which requires $\omega_{0}a/c_{l}$<<1 ($c_{l}=\sqrt{\kappa/\rho}$) and $\omega_{\max}a/c_{l}$<<1 ($\omega_{\max}=2\pi f_{\max}$, *f*_max_ represents the upper frequency limit of the study). As shown in Eq. (6), when the filling fraction is either extremely small or extremely large, the condition $\omega_{0}a/c_{l}$<<1 may not be satisfied. In such cases where the analytical model does not meet these conditions, the results are replaced by those calculated using the FEM.”

Regarding accuracy, we conducted a comparison with the finite element method. We found that the relative error of the results obtained by the transfer matrix method was not significant. The modifications in the results and discussions section were as follows: “To ascertain the accuracy of the TMM, we calculate the relative error (*α_t_*-*α_f_*)/*α_f_*, where *α_t_* represents the sound absorption coefficient obtained using the TMM, while *α_f_* represents the sound absorption coefficient obtained using the FEM. Within the frequency range of 10Hz to 10kHz, which is of our concern, the relative error for one-layer case is only 1.8%, while for two-layer case, it is 2.7%. Even for the three-layer case, which seems to have a larger error, the relative error is only 6.02%. As can be seen from the above results, the results calculated using the TMM are highly credible.”

This article mainly focuses on the advantage of the transfer matrix method in terms of fast calculation speed, which can quickly calculate a result. And it can be easily combined with genetic algorithms to obtain optimized results. In contrast, joint simulation calculations using COMSOL and MATLAB are very slow and, in most cases, fail to produce results. Therefore, there is not much discussion on the applicability, accuracy, and limitations of the model under different material and structural conditions.

We believe that these revisions have significantly strengthened the discussion of our theoretical model and have provided a more comprehensive and nuanced view of its capabilities and limitations. We appreciate your constructive feedback and hope that our revised manuscript meets your expectations.

3. The description of the complex relationship between the dipole resonance frequency (*ω*_0_) and the radius (*r*) is not sufficiently clear. I suggest that the authors further elaborate on the theoretical background of this relationship in Section 2 and provide practical examples to illustrate how it affects the results.

Thank you for your insightful feedback on the clarity of the description of the complex relationship between the dipole resonance frequency (*ω*_0_) and the radius (*r*). We understand that providing a more comprehensive theoretical background and practical examples to illustrate this relationship is crucial for the reader's understanding.

In the second part, the formula for the dipole resonance frequency is given as (*ω*_0_=*γc_s_*/*r*). On the surface, it appears that the dipole resonance frequency is inversely proportional to the radius *r*, but in fact, this is not the case. This is because γ in the formula is also related to the radius, and its expression is given in Eq. (6) ($\gamma=\left(\frac{8\left(1+\alpha_{1}^{2}\right)}{\left(\delta+\beta\right)\left(\left(1+\alpha_{1}^{2}\right)\ln\left(1/\alpha_{1}\right)+1-\alpha_{1}^{2}\right)}\right)^{0.5}$). In Eq. (6), the two parameters related to the radius *r* are *α*_1_ ($\alpha_{1}=\pi r^{2}/a^{2}$) and β ($\beta=\frac{1+\alpha_{1}}{1-\alpha_{1}}$). Therefore, we say that the relationship between the dipole resonance frequency and the radius *r* is complex, not simply a direct or inverse proportionality.

In the case study section, specifically in Figure 6(c), we made the following modifications: “The influence of the radius of the inclusions on the absorption is depicted in Fig 6(c). Other parameters are the same as those in Fig 5(a). It can be seen that the influence of the radius on the absorption performance is obvious and complicated. This can be explained as follows: From Equations (3)-(5) and (10), it can be deduced that the dipole resonance frequency affects the equivalent density, which in turn influences the sound absorption coefficient. Similarly, based on Equations (2), (11), and *α*_1_=π*r*^2^/*a*^2^, it is evident that the radius impacts the equivalent modulus, which further affects the sound absorption coefficient.”

We believe that these revisions have significantly strengthened the manuscript by providing a more detailed and comprehensive understanding of the *ω*_0_-*r* relationship and its impact on our results. We appreciate your constructive feedback and hope that our revised manuscript meets your expectations.

4. Although the impedance mismatch analysis presented in Fig. 7 provides qualitative insights, it lacks quantitative analysis. I recommend that the authors supplement relevant parameters to clarify the conditions under which reflected waves are trapped near the periodic multi-layer structure and quantitatively correlate this with impedance mismatch. Providing more detailed numerical simulations or experimental data to validate this phenomenon would also strengthen the analysis.

Thank you for your insightful comments regarding our manuscript. We appreciate your feedback on the impedance mismatch analysis presented in Fig. 7. We acknowledge the importance of providing a quantitative analysis to complement the qualitative insights already discussed.

We have made the following modifications in the corresponding paragraphs as follows：“To further understand the underlying mechanism of broadband sound absorption, the displacement field and the energy dissipation density of the sound-absorbing structure are studied at the peak frequencies of 1960Hz (*α*=0.684), 4340Hz (*α*=0.994) and 7960Hz (*α*=0.999), respectively. As shown in Fig 7(b)-(d), the acoustic wave is incident normally from the left side of the structure. The displacement field diagram is on the left side, while the energy dissipation diagram is on the right side. Overall, the displacement amplitude is large, and the corresponding level of energy dissipation is also high. When the excitation frequency is 1960Hz, the maximum displacement amplitude is located in the upper and lower PDMS of the right scatterer, and the corresponding energy dissipation is concentrated in these two places. At the second peak (4340 Hz), the areas with the highest displacement amplitude are the PDMS above and below the left scatterer, in which energy dissipation is also concentrated. At the third peak (7960 Hz), energy dissipation is also concentrated above and below the left scatterer. However, for the third peak, the dissipation of energy occurs closer to the incident side compared with the second peak. From these phenomena, we can observe that lower frequencies are more difficult to dissipate, and their dissipation positions are closer to the steel plate side. As the frequency increases, energy becomes easier to dissipate and occurs closer to the incident side. In addition to the fact that the incident longitudinal waves are converted into transverse waves under the influence of the periodic cylinders and subsequently dissipated by the PDMS[14], another important reason is that, the acoustic impedance at the incident side is relatively matched, while, at the steel plate side, the acoustic impedance is mismatched, causing further dissipation. We can provide a quantitative explanation for this from Fig 8. As evident from Fig 8, at 1960 Hz, the normalized surface acoustic resistance *Z_r_* is 2.56, and the normalized surface acoustic reactance *Z_i_* is -1.52, whereas at 4340 Hz, these values are 1.04 and 0.16, respectively, and at 7960 Hz, they are 1.05 and 0.05. It is well-known that the requirements for perfect sound absorption are met when *Z_r_* = 1 and *Z_i_* = 0. This explains why near-perfect sound absorption is achieved at 4340 Hz and 7960 Hz, whereas at 1960 Hz, the corresponding conditions are not met, resulting in a sound absorption coefficient of only 0.684. Furthermore, as seen in Fig 8, the normalized surface acoustic impedance within the range from 1960 Hz to 10 kHz approaches the conditions for perfect sound absorption, enabling efficient broadband sound absorption within this frequency range.

Fig 8. The acoustic resistance (blue lines) and reactance (red dotted lines) of the optimization case in Fig 7.”

We believe that these additions will significantly improve the clarity and depth of our analysis, addressing your concerns effectively. Thank you once again for your valuable feedback.

5. Optimization using the genetic algorithm is a highlight of the manuscript, but the limitations of this optimization approach are not adequately discussed. I suggest the authors include a discussion on the potential limitations of the optimization design and reference the latest relevant research to further emphasize the novelty of their work. This would help readers better understand the uniqueness and practical value of this study.

Thank you for highlighting the importance of discussing the limitations of the genetic algorithm optimization approach used in our manuscript. You are correct that a thorough examination of the potential limitations is crucial for readers to fully appreciate the novelty and practical value of our work.

In response to your suggestion, we have made the following revisions to our manuscript:

Reference to Relevant Research: To further emphasize the novelty of our work, we have incorporated references to the latest relevant research on genetic algorithm optimization and other optimization techniques. These references provide context for our study and highlight how our approach differs from and builds upon previous work. By situating our work within the broader research landscape, we hope to better illustrate its unique contributions and practical implications. The corresponding modifications are as follows: “The geometric parameters of acoustic coatings significantly impact the absorption coefficient, usually exhibiting a nonlinear relationship. Hence, directly achieving broadband and superior sound absorption performance through experience or parametric scanning poses significant challenges. Various optimization Algorithms, including Nelder-Mead method, GA, Simulated Annealing (SA), and Particle Swarm Optimization (PSO), have garnered widespread adoption in acoustic applications [43-46]. To achieve enhanced absorption performance within the specified frequency range, we employ a global, fast and robust GA [22, 23] for optimization.”

Discussion of Limitations: We have discussed the potential limitations of the genetic algorithm optimization approach as follows: “Despite the numerous advantages of GA, it also possesses a significant number of drawbacks, including the computational cost associated with large search spaces, the sensitivity to parameter settings, and the potential for getting stuck in local optima. We also acknowledge that while GA has proven effective in our study, there may be other optimization techniques that could offer advantages in certain scenarios.”

Clarification of Novelty and Practical Value: Throughout the revised manuscript, we have made a concerted effort to clarify the novelty and practical value of our study. We have emphasized how our optimization design addresses specific challenges in the field and how the results of our work can inform future research and applications. By highlighting these aspects of our work, we hope to make a compelling argument for its significance and impact.

We appreciate your feedback and believe that these revisions have significantly improved the manuscript. By discussing the limitations of the genetic algorithm optimization approach and referencing relevant research, we have provided readers with a more complete and nuanced understanding of our work. We hope that these changes will help readers better appreciate the uniqueness and practical value of our study.

Reviewer #5: The comments have been well addressed and incorporated into revised the manuscript, and the current version of the article is now acceptable.

Thank you for your positive feedback and for acknowledging that the comments have been well addressed and incorporated into the revised manuscript. We are delighted to hear that the current version of the article is now acceptable to you.

We are grateful for your constructive suggestions and thorough review of our work. Your input has been invaluable in helping us improve the quality and clarity of our manuscript.
